# Inconspicuous Consumption: Uncovering the Molecular Pathways behind Phagocytosis

**DOI:** 10.1371/journal.pbio.0040190

**Published:** 2006-05-09

**Authors:** Mason Inman

Eating at an Ethiopian restaurant might give one an appreciation for the process of phagocytosis, a widespread method immune cells use to ingest particles. To dine using injera, the spongy Ethiopian flatbread, you form a pocket of bread around the food that you pinch off and, with practice, stuff cleanly into your mouth. Similarly, when cells ingest small particles, viruses, or other smaller cells by phagocytosis, they extend protrusions called pseudopods around their target. The pseudopods then seal around the object, and a bit of the cell membrane that encases the object buds off and travels inside the cell.

Figuring out which molecules are involved in coordinating the fast-moving, dynamic process of phagocytosis has been difficult. But using a sensitive, high-resolution technique called fluorescence resonance energy transfer (FRET), a new study reveals the patterns of protein activities that help regulate phagocytosis. Peter Beemiller, Adam Hoppe, and Joel Swanson show that two stages of phagocytosis—the extension of pseudopods and the closure around an object—each require activation of specific proteins, members of the ADP ribosylation factor (ARF) family, which are major regulators of the budding of vesicles, or membrane-bound sacs, from internal organelles. The researchers also found that an enzyme called phosphatidylinositol-3′-kinase (PI-3K) plays a key role in organizing these two stages of phagocytosis.

Key to the discovery was a technique called FRET. Though the technique was first described nearly 50 years ago, it has recently been greatly refined and has experienced a renaissance. The technique relies on the ability of one fluorescent molecule to excite another fluorescent molecule, which emits light when the two molecules are very close to each other. Thus, when exciting the first fluorescent molecule lights up the second one, it signals that the two fluorescent molecules are within tens of angstroms—practically on top of each other.

To use FRET for picking apart the pathways involved in phagocytosis, the researchers made mouse phagocytes that expressed engineered proteins—versions of ADP ribosylation factors (ARFs) 1 and 6 had fluorescent protein attached, and fluorescent marker proteins that would bind to either ARF1 or ARF6, when they had been activated inside the cell. Binding of the activated fluorescent ARFs to the fluorescent markers could be detected via FRET. Thus, the researchers could see when and where ARF1 or ARF6 were active in the cell during phagocytosis.

The researchers found that pseudopods extending around a particle activated ARF6 at their tips. Then as the cell more fully enveloped a particle, ARF6 turned off, and activated ARF1 appeared throughout the pseudopod until it closed around the particle. The discovery of a role for ARF1 was unexpected, since previous studies suggested this protein is not involved in phagocytosis. Also, the researchers found that inhibiting PI-3K prevented cells from switching between these two phases of phagocytosis, and caused them to stop halfway without having closed around the particles.

It's still not entirely clear what role each of the ARFs plays. ARF6 may regulate how the cell supplies more membranes to the area of phagocytosis, so that the cell can expand its surface area there and engulf particles. ARF1, on the other hand, plays a key role in protein trafficking, and so may regulate which proteins migrate to the site of phagocytosis. But finding that phagocytosis has distinct phases, regulated by phospholipids in the membrane, is an important step toward sorting out how thousands of receptors can be coordinated in time and space to make a phagosome.

## 

**Figure:**
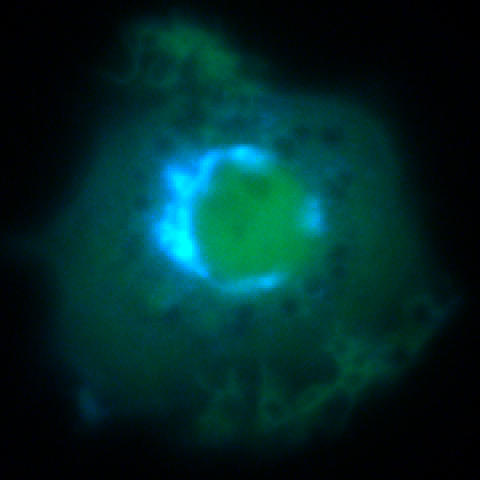
Fluorescent proteins localize the guanosine 5'-triphosphate hydrolase ARF in the Golgi apparatus of a living macrophage. FRET studies revealed ARF activation in the Golgi and in the formation of phagosomes.

